# A systematic review of peste des petits ruminants in Bangladesh: Epidemiology, diagnostics, risk factors, vaccination, and gaps toward eradication

**DOI:** 10.14202/vetworld.2026.2358-2370

**Published:** 2026-06-10

**Authors:** Sheuly Akter, Mst Sogra Banu Juli, Maksudur Rashid, Shukes Chandra Badhy, Shuvo Mazumder, Md Mostofa Kamal

**Affiliations:** 1 Livestock Research Institute, Mohakhali, Dhaka, Bangladesh; 2 The University of Queensland, Queensland Alliance for Agriculture and Food Innovation (QAAFI), St Lucia, Queensland 4067, Australia; 3 Charles Sturt University, Wagga Wagga, New South Wales, Australia; 4 Central Disease Investigation Laboratory, Dhaka, Bangladesh

**Keywords:** Bangladesh, diagnostics, epidemiology, peste des petits ruminants, risk factors, seroprevalence, vaccination, viral diseases

## Abstract

**Background and Aim:**

Peste des petits ruminants (PPR) is a highly contagious transboundary viral disease that severely impacts small ruminant production systems, particularly in low- and middle-income countries. In Bangladesh, where goats and sheep are critical to rural livelihoods, PPR remains endemic and poses substantial economic and food security challenges. This systematic review aimed to synthesize current evidence on the epidemiology, diagnostic approaches, risk factors, vaccine performance, and existing knowledge gaps to support national and global eradication efforts.

**Materials and Methods:**

A systematic review was conducted following Preferred Reporting Items for Systematic Reviews and Meta-Analyses (PRISMA) 2020 guidelines. A total of 930 records were identified through PubMed and Google Scholar, of which 22 eligible studies published between 2001 and April 2025 were included after rigorous screening. Data were extracted using a standardized framework and synthesized qualitatively due to heterogeneity in study designs, diagnostic methods, and outcome measures.

**Results:**

The majority of studies (96%) focused on goats and sheep, highlighting their primary role in disease epidemiology. Reported prevalence varied widely (2.18%–42.26%), with higher susceptibility consistently observed in goats. Seroprevalence studies indicated substantial viral circulation across regions, with notable geographic disparities in research coverage. Diagnostic methods included clinical assessment, competitive enzyme-linked immunosorbent assay, and reverse transcription polymerase chain reaction, with limited integration of advanced molecular tools. Molecular characterization confirmed the exclusive circulation of Lineage IV strains, closely related to those from neighboring countries, indicating transboundary transmission. Key risk factors included age, seasonality, breed, animal movement, and vaccination status. Co-infections with bacterial, viral, and parasitic pathogens were frequently reported, complicating diagnosis and disease management. The locally produced vaccine demonstrated strong immunogenicity, with seroconversion rates exceeding 91% and fieldlevel protection lasting up to 12 months; however, vaccination coverage remains uneven across regions.

**Conclusion:**

Despite progress in surveillance and vaccination, significant gaps persist in multi-species epidemiology, advanced diagnostics, co-infection management, and Differentiation of infected from vaccinated animals (DIVA)-compatible vaccine development. Strengthening integrated surveillance, improving vaccination coverage, and adopting rapid molecular diagnostics are critical to achieving PPR eradication targets in Bangladesh by 2030.

## INTRODUCTION

Peste des Petits Ruminants (PPR), often dubbed the “plague of small ruminants,” has a devastating impact in low-income countries where goats and sheep are integral to rural livelihoods. In Bangladesh, goats commonly called the “poor man’s cow” are prized for their low maintenance, high fertility, and economic resilience. Bangladesh is home to approximately 27.291 million goats, 3.981 million sheep [1] and approximately 0.12 million [2] wild small ruminant which are deer, gazelle and others. Over 98% small ruminant population are managed by smallholder, marginal, or landless farmers [3]. PPR is a highly contagious, transboundary viral disease caused by a morbillivirus closely related to rinderpest. The virus has a negative-sense, single-stranded RNA genome encoding six structural (N, P, M, F, H, and L) [4] and two non-structural (C and V) proteins [5]. Genetically, PPR virus (PPRV) is classified into four distinct lineages (I–IV) [5, 6] differentiated by nucleoprotein (N) and fusion (F) gene sequences. Lineages I and II are found in West Africa, Lineage III in East Africa [7], and Lineage IV, the only lineage circulating in Bangladesh, is prevalent in Asia and parts of the Middle East [5, 7].

Clinically, PPR manifests with high fever, nasal and ocular discharge, oral erosions, diarrhea, respiratory distress, and often death due to secondary complications [8–10]. The virus exhibits marked lymphotropism and induces immunosuppression characterized by leukopenia and impaired humoral immune responses [11, 12]. While goats and sheep are primary hosts, emerging reports suggest an expanding host range, including clinical cases in camels [13, 14] and subclinical infections in cattle [15–17] and buffalo [18]. However, virus shedding and transmission from these atypical hosts remain unconfirmed. Transmission primarily occurs through direct contact with infected secretions and excretions [19]. Recovered animals are generally considered to develop long-term immunity without becoming carriers [20]. The first confirmed outbreak of PPR in Bangladesh occurred in 1993 in Meherpur district and was verified by an international reference laboratory [21]. Sero-epidemiological studies in Bangladesh have reported substantial exposure to PPRV among goats, indicating widespread circulation of the virus [22]. Since then, the disease has remained endemic. PPR causes substantial economic losses in South Asia, with annual losses estimated in the billions of United States dollars [23]. The annual economic loss due to PPR in Bangladesh is estimated at approximately 25 million United States dollars [24], although the exact national burden remains uncertain due to limited economic assessments [25].

To address this issue, the Food and Agriculture Organization and the World Organization for Animal Health launched the Global Strategy for the Control and Eradication of PPR in 2015, targeting global eradication by 2030 [23]. Bangladesh is an active participant in this program. Bangladesh initially aimed to eradicate PPR by 2026 through nationwide vaccination and surveillance; the revised strategy, aligned with the Food and Agriculture Organization–World Organization for Animal Health global program, extends verification and intensified surveillance through 2028, ahead of global certification by 2030 [26]. As of 2025–2026, South Asia remains in the active control stage. Although Bangladesh targeted reaching Stage 3 by 2025, this has not yet been achieved; however, substantial progress continues through strengthened implementation of the national strategic plan and risk-based approaches. In 2001, local vaccine production was initiated to meet domestic demand. This not only aids disease control but also reduces antimicrobial use and conserves foreign exchange. Despite numerous studies on epidemiology, seroprevalence, molecular characteristics, and vaccination strategies, to our knowledge only one meta-analysis on prevalence has been published [27].

Despite the growing body of literature on PPR in Bangladesh, several critical gaps remain inadequately addressed. First, most studies are geographically clustered and predominantly focused on goats, with limited representation of sheep, large ruminants, and wildlife, restricting a comprehensive understanding of multi-species epidemiology. Second, although conventional diagnostic tools such as enzyme-linked immunosorbent assay and reverse transcription polymerase chain reaction (RT-PCR) are widely used, the adoption of advanced molecular techniques and rapid field-deployable diagnostics remains limited, hindering early detection and real-time surveillance. Third, the role of co-infections and their interaction with PPRV pathogenesis has received minimal attention, despite increasing evidence of their impact on disease severity and management outcomes.

In addition, socio-economic determinants, farmer awareness, and husbandry practices are underexplored, even though these factors significantly influence disease transmission and control. There is also a lack of robust longitudinal and nationwide data on vaccine effectiveness, coverage variability, and herd immunity thresholds under field conditions. Furthermore, the absence of differentiating infected from vaccinated animals-compatible vaccine strategies limits post-vaccination surveillance and disease monitoring. Finally, economic burden assessments and policy-driven evaluations of eradication programs remain insufficient, constraining evidence-based decision-making for national control strategies.

This systematic review was conducted to comprehensively synthesize available evidence on PPR in Bangladesh with a focus on epidemiology, diagnostic approaches, molecular characteristics, risk factors, and vaccine performance. The study aims to critically evaluate the distribution and burden of PPR across regions and species, assess the effectiveness and limitations of currently used diagnostic and surveillance methods, and examine host, environmental, and management-related risk factors influencing disease occurrence.

In addition, this review aims to identify knowledge gaps in multi-species epidemiology, co-infection dynamics, socio-economic determinants, and vaccination strategies, including the need for improved surveillance tools and innovative vaccine approaches. By integrating findings from diverse studies, this review seeks to provide a consolidated evidence base to support risk-based interventions, strengthen national surveillance systems, and inform policy frameworks aligned with global eradication targets. Ultimately, the study contributes to advancing strategies for sustainable PPR control and eradication in Bangladesh by 2030.

## MATERIALS AND METHODS

### Ethical approval

This study was a systematic review and did not involve any direct experimentation on animals or humans. All data were obtained from previously published studies, reports, and publicly accessible databases. Therefore, ethical approval was not required for this study.

The review was conducted in accordance with the Preferred Reporting Items for Systematic Reviews and Meta-Analyses (PRISMA) 2020 guidelines to ensure transparency, methodological rigor, and reproducibility. All included studies were assumed to have been conducted in compliance with relevant institutional and national ethical standards for animal research.

No live animals were handled, no biological samples were collected, and no interventions were performed during the course of this study. Accordingly, issues related to animal welfare, consent, or ethical clearance are not applicable to this review.

However, due consideration was given to the ethical use and citation of published data. All sources were appropriately acknowledged, and no data were manipulated or misrepresented.

### Study period and location

The literature search, study selection, data extraction, data synthesis, and data analysis were carried out between April and September 2025 at the PPR Vaccine Production Section, Livestock Research Institute, Mohakhali, Dhaka, Bangladesh.

### Protocol registration and reporting standards

**Figure 1:**
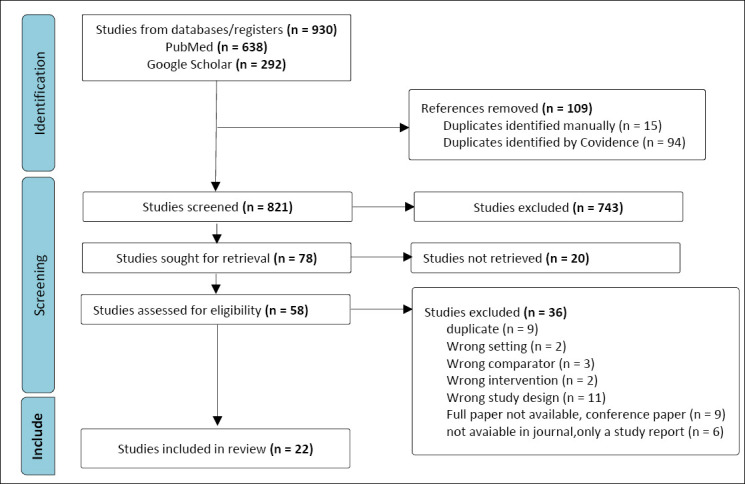
The steps from the Preferred Reporting Items for Systematic Reviews and Meta-Analyses (PRISMA) utilized for this systematic review.

This review was not prospectively registered, as it represents a comprehensive descriptive synthesis rather than an intervention-based systematic review with predefined effect measures. However, the study was conducted in full compliance with PRISMA 2020 guidelines, and the flow diagram is presented in Figure 1.

### Search strategies and eligibility criteria

A comprehensive literature search was conducted using two electronic databases, PubMed and Google Scholar, to identify relevant articles published in English from 2001 to April 2025. The search strategy employed the following keywords: “Peste des Petits Ruminants OR PPR OR Goat Plague OR Kata OR PPR Vaccine OR PPR Prevalence AND Bangladesh.” Original studies conducted in Bangladesh on goats and/or sheep reporting PPR-related outcomes were included, whereas review articles, non-original articles, non-English studies, and studies lacking primary data were excluded.

### Review question and eligibility framework

The review objective was structured using the population, exposure, comparator, and outcome (PECO) framework (Table 1). The population comprised goats and sheep in Bangladesh; exposure was PPRV infection and associated risk factors; comparators included non-infected animals or exposure groups; and outcomes included PPR prevalence, diagnostics, molecular epidemiology, and vaccine performance. Eligibility criteria and outcome measures were defined consistently with this framework.

**Table 1:** The PECO framework applied to define the research question, eligibility criteria, and outcome measures.

PECO component	Definition in this review	Corresponding eligibility criteria	Extracted outcome measures
Population	Domestic small ruminants (goats and sheep) in Bangladesh	Studies conducted in Bangladesh involving goats and/or sheep	Species-specific prevalence, morbidity, mortality
Exposure	PPRV infection and associated epidemiological risk factors	Studies reporting confirmed or suspected PPR using recognized diagnostic methods	Seroprevalence, incidence, risk factor associations
Comparator	Non-infected animals or comparison across exposure categories (age, sex, region, management, vaccination status)	Studies including comparative groups or stratified analyses	Odds ratios, relative risks, subgroup comparisons
Outcome	Burden and epidemiological characteristics of PPR	Studies reporting epidemiological, diagnostic, molecular, or vaccine-related data	Prevalence/seroprevalence, diagnostic performance, molecular lineages, vaccine efficacy or effectiveness

### Information sources and search strategy

A comprehensive literature search was conducted in the electronic databases PubMed (National Center for Biotechnology Information) and Google Scholar to identify relevant English-language articles published between 2001 and April 2025. These databases were prioritized because they comprehensively index peer-reviewed veterinary and PPR-related literature relevant to Bangladesh. To minimize potential selection bias, this search was complemented with gray literature sources, including World Organization for Animal Health World Animal Health Information System reports, Food and Agriculture Organization/World Organization for Animal Health regional updates, and Department of Livestock Services annual reports. The final search was performed on 30 April 2025. Only open-access, English-language original research articles published from 2001 to April 2025 were considered eligible. Review articles, conference proceedings, and book chapters were excluded to ensure methodological rigor and originality.

### Study selection and screening process

Two independent reviewers screened each article against predefined inclusion and exclusion criteria (Supplementary Table 1). Discrepancies were resolved by a third reviewer. A total of 84 articles underwent full-text review conducted independently by two reviewers. After final screening, 62 articles were excluded, and 22 studies met the eligibility criteria and were included in this review following PRISMA 2020 guidelines, and the selection process is summarized in Figure 1 [28].

### Data extraction and data management

Data from the 22 included studies were extracted using a standardized Excel-based template, capturing key variables such as article title, authors, publication year, study location, diagnostic methods, prevalence or seroprevalence rates, PPRV lineage, identified risk factors, morbidity and mortality rates, and vaccine effectiveness.

### Data synthesis and analysis strategy

A qualitative synthesis was performed due to heterogeneity in study design, outcomes, and diagnostic methods. Prevalence, risk factors, molecular data, and vaccine performance were summarized descriptively in tables and figures. A meta-analysis was not performed because the included studies exhibited substantial variability in study design, sample size, diagnostic methods, and outcome measures, which precluded meaningful quantitative pooling. Descriptive statistics were used to summarize the data, with results presented in tables and graphical formats for clarity.

### Assessment of heterogeneity

Heterogeneity was assessed based on clinical factors (species, region, management), methodological differences (study design, diagnostics), and statistical variation. Subgroup and sensitivity analyses were performed descriptively to explore variability and ensure robust interpretation.

### Publication bias assessment

Publication bias was not formally assessed due to study heterogeneity and lack of quantitative pooling. However, comprehensive database searches and reference screening were conducted to minimize potential bias.

### Software and tools

Articles were imported into Zotero (version 7.0.15.0) for reference management and exported as an RIS file to Covidence (https://www.covidence.org/) for systematic screening.

### Handling of missing data and disagreements

Missing or unclear data were addressed by contacting study authors; unresolvable data were recorded as “not reported.” Reviewer disagreements at all stages were resolved by consensus or, when necessary, by a third reviewer.

### Protocol deviations and amendments

No formal protocol registration was performed. Minor methodological amendments, including refinement of search terms and pilot testing of data extraction forms, were documented and did not affect the review objectives.

### Data availability and transparency statement

All extracted data, standardized tables, search strategies, and risk-of-bias assessments are provided in the Supplementary Materials. Additional information is available from the corresponding author upon reasonable request.

## RESULTS AND DISCUSSION

### Study characteristics and species distribution

**Figure 2:**
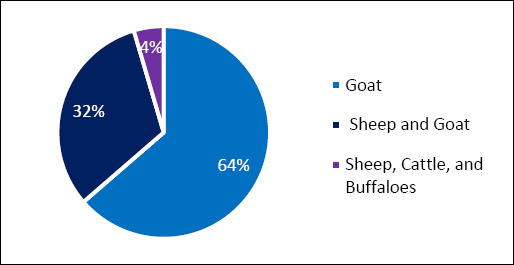
Pie chart showing the frequency distribution of PPR-related studies by species included in this review.

A total of 22 studies were included, conducted across different regions of Bangladesh, exhibiting considerable heterogeneity in study design, target populations, and sample sizes. Most studies focused on small ruminants 96% (exclusively 64% on goats and 32% on both sheep and goats). Only one study (4%) was related to large ruminants such as cattle and buffalo (Figure 2).

This emphasis reflects the documented role of goats and sheep as the most affected species in Bangladesh. Goats (64%) were most studied, likely reflecting their greater susceptibility to PPRV, faster disease progression, and substantial economic importance in smallholder farming systems. Only 4% of studies focused on sheep, cattle, or buffalo, likely because these species develop subclinical PPRV infections without significant transmission potential [16, 17, 25]. The role of cattle in PPRV epidemiology remains unclear, although several studies [17, 26] report spillover infections from small ruminants. It has been suggested that cattle could serve as sentinel species during small ruminant mass vaccination campaigns [17]. Key findings from the individual studies categorized according to predefined criteria are summarized in tabular form and provided as a supplementary file (Supplementary Table 2).

### Geographical distribution and epidemiology of PPR in Bangladesh: prevalence, morbidity, and mortality

The majority of studies were conducted in Mymensingh Division (n = 16; 72.72%), followed by Rajshahi (n = 14; 63.63%) and Rangpur (n = 13; 59%). A moderate proportion was carried out in Dhaka Division (n = 11; 50%), whereas relatively fewer studies were reported from Khulna (n = 4; 18.18%) and Chattogram (n = 4; 18.18%). The lowest numbers of studies were documented in Sylhet (n = 2; 9.09%) and Barishal (n = 2; 9.09%).

**Figure 3:**
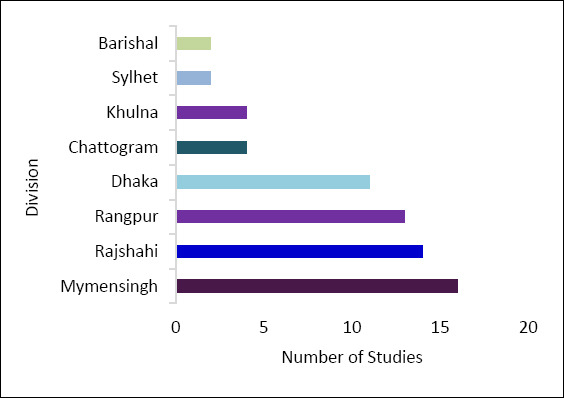
Bar chart showing the geographical distribution of PPR-related studies conducted in Bangladesh.

Geographically, PPR-related research exhibited significant disparities. Most studies originated from Mymensingh, Rajshahi, and Rangpur divisions, likely reflecting the proximity of key research institutions such as Bangladesh Agricultural University in Mymensingh, higher small ruminant population densities, and logistical feasibility. In contrast, Dhaka showed moderate research activity, while Khulna and Chattogram were underrepresented. Sylhet and Barishal divisions had the fewest studies, highlighting notable geographic gaps and the need for more inclusive national surveillance efforts to obtain a comprehensive epidemiological picture and improve control strategies (Figure 3).

A total of six studies (27.27%) reported PPR prevalence across different regions of Bangladesh. Consistently higher prevalence was observed in goats compared to sheep, corroborating species susceptibility differences [29, 30]. Reported prevalence ranged from 2.18% to 42.26%, with the highest prevalence (42.26%) in goats reported from January–April 2018 in Gangachara, Rangpur [31]. During March 2010 to January 2011, PPR prevalence in goats was reported at 20.57% in Rajshahi [32]. From January 2019 to March 2021, 18.9% prevalence among goats was found in slaughterhouses in Mymensingh Division [10], highlighting potential sources of horizontal PPRV transmission between markets and farms and underscoring the need for improved biosecurity in animal markets.

From 2010 to 2012, an overall prevalence of 14.68% was reported in Rajshahi, Mymensingh, and Chattogram divisions, with 94.8% of cases in goats and 5.2% in sheep [33]. A 9.32% prevalence was reported in Rangpur Sadar, mainly affecting goats (81.82%) compared to sheep (18.18%) [34]. Recent prevalence estimates indicated 21.3% in Jashore during 2023–2024 [35]. Conversely, a lower prevalence of 2.18% in goats was recorded in Sujanagar, Sathia, and Bera upazilas of Pabna district from April–December 2010 [36]. Reported PPR prevalence varied widely, with studies providing estimates ranging from 2.18% in Pabna district to 42.26% in Gangachara, Rangpur. Importantly, slaughterhouses were highlighted as potential transmission hotspots [10], underscoring the need for improved biosecurity in animal markets. These findings emphasize regional variation in disease burden and the necessity for targeted vaccination and outbreak response.

Seroprevalence of PPRV was evaluated in 18.18% of the studies using serological assays, predominantly competitive enzyme-linked immunosorbent assay kits targeting either the hemagglutinin (H) antigen or the nucleoprotein of the virus. Details on the number of animals sampled by location, sex, and species are summarized in Supplementary Table 2. Considering species, the highest seroprevalence was recorded in buffalo (42.36%), followed by 27% in sheep and 25% in goats. Sample sizes used for seroprevalence varied, ranging from 124 animals to 434 animals. Conducting a geographic analysis of seroprevalence across six districts of Bangladesh; Chuadanga, Sirajganj, Thakurgaon, Satkhira, Jhenaidah, and Chattogram, by sampling both sheep and goats up to one year from 2016–2017, the highest seroprevalence was reported (56.92%) in Satkhira, while the lowest was observed (30.79%) in Chattogram [37]. Seroprevalence estimates varied by species, with buffalo exhibiting the highest overall seropositivity (42.36%) despite limited data on large ruminants. Many studies lacked clear criteria for animal selection, underscoring the need for more rigorous surveillance, including wild ruminants, to assess their role in PPR epidemiology and wildlife conservation [38, 39]. Outbreaks in wild ungulates are likely spillover events from domestic small ruminants, highlighting the importance of monitoring the livestock–wildlife interface. Differences in seroprevalence between goats and sheep may be influenced by unreported factors such as management practices and flock demographics [40, 41]. Notably, 18.18% of seroprevalence studies were linked to outbreak investigations, potentially biasing prevalence estimates.

Unfortunately, very few studies reported morbidity and mortality in goats and sheep caused by PPR. Only 13.63% of studies reported morbidity and mortality, among which the highest fatality was recorded in Jhenaidah (93.75%, 75 out of 80) in small ruminants [37]. In goats from affected households during a PPR outbreak in Mymensingh district, morbidity and mortality rates were recorded at 74.13% and 54.83%, respectively [22]. In contrast, in Chuadanga farms affected during November–December 2014, a low morbidity rate (15.49%) but high case-fatality rate (54.54%) was observed [42]. These findings highlight the variability of PPR burden across districts and time periods, reflecting differences in surveillance, reporting, husbandry practices, and possibly virus virulence.

### Diagnostic approaches and molecular characterization of PPRV Lineage IV in Bangladesh

**Figure 4:**
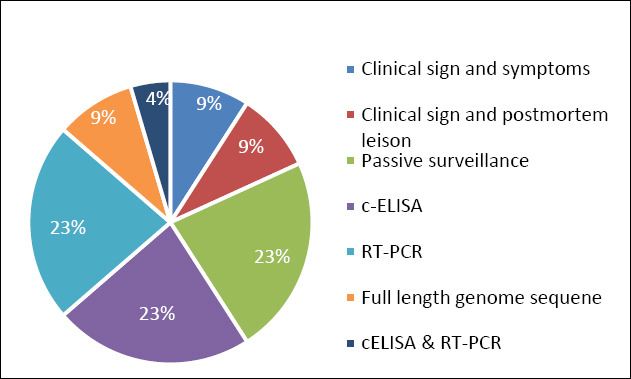
Pie chart illustrating the most commonly used diagnostic approaches for PPR in Bangladesh.

This review highlights the use of diverse diagnostic methodologies to detect PPRV infection using clinical, pathological, molecular, and serological approaches. Molecular and serological methods ([RT-PCR] and enzyme-linked immunosorbent assay, each 23%) and passive surveillance (23%) are the most frequently employed diagnostic approaches for PPR in Bangladesh (Figure 4). In contrast, reliance on clinical signs (9% each, with and without postmortem lesions) and genomic techniques remains comparatively limited, while combined diagnostic methods (enzyme-linked immunosorbent assay and RT-PCR) are rarely used (4%).

Among the studies reviewed, 9% reported gene sequencing to characterize circulating PPRV lineages (Supplementary Table 2). All isolates originated from goats and/or sheep. Lineage IV strains were consistently identified using RT-PCR targeting partial nucleoprotein and fusion genes from postmortem samples collected between 2008 and 2012 [43], with two unique nucleoprotein substitutions (K423Q and E426G) also reported in Chinese PPRV isolates. Goats from Mymensingh were analyzed for nucleoprotein gene sequences (eight samples), showing high homology with strains from India, China, Dubai, Iran, Pakistan, Turkey, Sudan, and Mongolia, indicating ongoing evolution and transboundary circulation within Lineage IV [10]. Complete genome sequencing of isolates collected between 2008 and 2020 further confirmed clustering within Lineage IV, with close genetic relationships to strains from China, Tibet, and India [44, 45]. High nucleotide identity (>99%) among Bangladeshi isolates and strong similarity to Indian strains underscore the importance of cross-border transmission dynamics and the need for coordinated regional surveillance. Emerging rapid molecular techniques such as Reverse transcription loop-mediated isothermal amplification, Reverse transcription recombinase polymerase amplification, and SYBR Green RT-qPCR [46–48] offer high sensitivity and field applicability but remain underutilized in Bangladesh.

### Host and environmental risk factors

**Figure 5:**
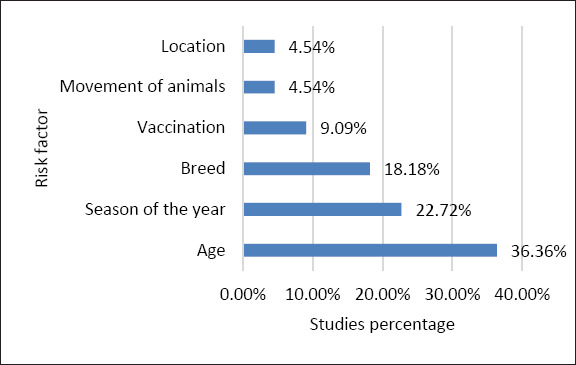
Column chart illustrating studies related to risk factors identified for PPR disease prevalence in Bangladesh.

Among the eligible studies, 95.54% reported significant host- and environment-related risk factors associated with PPRV in Bangladesh. Host-related determinants exert the greatest influence, with age accounting for 36.36% of reported associations, followed by seasonal variation (22.72%) and breed (18.18%), whereas vaccination (9.09%), animal movement (4.54%), and geographic location (4.54%) were comparatively less represented (Figure 5). These results indicate that intrinsic host characteristics and temporal factors are primary drivers of PPR epidemiology, while management and spatial determinants play a secondary role.

Multiple studies (36.36%) consistently identified age as a significant risk factor, with younger animals exhibiting greater susceptibility to PPRV infection. In 4.54% of studies, an elevated risk was reported in goats aged 5–8 weeks during July 2006 in Mymensingh district [22], and 4.54% of studies found increased seropositivity in animals older than 4–6 months [49]. In 9.09% of studies, higher infection rates were noted in goats older than 3 months [36, 50], and 9.09% revealed the highest susceptibility in the 7–12 months age group, with rates of 40.91% and 48.45%, respectively [31, 32, 36]. Another 4.54% of studies reported a 31.06% susceptibility in young goats, although age was not specified [37].

Breed was another important risk factor, primarily comparing goat breeds in 18.18% of studies. Three studies compared Black Bengal with Jamunapari goats [31, 32, 36], while one study compared Black Bengal with exotic breeds [37]. The highest susceptibility was observed (72.32%) in Black Bengal goats [34], while exotic breeds showed the lowest susceptibility (9.68%) [32]. Disease incidence in both sheep and goats was highest during the monsoon season in 9.09% of studies, with reported prevalence rates of 27.6% and 26.7%, respectively [24, 33]. These studies applied geographically weighted regression and Chi-square statistical analyses, underscoring spatial and seasonal clustering during humid months, typically from June to August. In contrast, 4.45% of studies, employing a spatial scan statistic (Bernoulli model), reported peak disease prevalence in goats during winter (30%) and monsoon (28%) seasons [49], while 4.5% of studies reported higher prevalence during the rainy season.

Vaccinated animals exhibited significantly greater resistance to PPRV compared to non-vaccinated counterparts [22, 51]. Animal movement was identified as a significant risk factor for PPRV spread, with road length showing a statistically significant association (p = 0.03) [24]. Studies that did not account for livestock movement patterns or biosecurity practices may underestimate the risk of transboundary or local transmission, introducing potential bias in risk assessment. Limited reporting on movement controls, quarantine measures, and farm-level biosecurity further increases uncertainty in interpreting prevalence and outbreak data. These gaps highlight the need for integrated surveillance that considers animal mobility, transportation routes, and biosecurity compliance to reduce bias and improve the accuracy of risk estimation. Geographic location was also highlighted as a critical risk factor, with the north-west, north-east, and south-east regions of Bangladesh exhibiting the highest susceptibility to outbreaks.

Key risk factors included age (3–12 months), breed (Black Bengal goats), and seasonality (monsoon and winter), suggesting climatic stress exacerbates susceptibility. Vaccinated animals exhibited significantly greater resistance to PPRV compared to non-vaccinated counterparts [22, 51]. While females generally showed higher seropositivity, results were inconsistent. These findings advocate for risk-based, spatially informed surveillance and control.

### Socio-economic status, rearing practices, and farmer knowledge of PPR

Only 13.63% of the reviewed studies explored socio-economic conditions, rearing practices, and awareness of PPR among goat farmers, with data primarily from Panchagarh, Jamalpur, and Kurigram districts. Annual income of farmers varied, with 36.9% earning less than 10,000 Bangladeshi Taka (BDT), 52.5% earning between 10,000–20,000 BDT, and only 10.6% earning above 20,000 BDT. In terms of husbandry, 67.3% of farmers followed a semi-intensive system, 24.1% practiced free-range, and only 8.5% used intensive methods. Socio-economic and husbandry practices were explored in 9.09% of studies from regions including Panchagarh, Jamalpur, Kurigram, and Sylhet’s Haor areas. These studies shed light on critical human factors influencing PPR control [52, 53]. For example, 57% of goat owners were female, highlighting women’s significant role in small ruminant production and emphasizing the need for gender-sensitive awareness and vaccination programs. Over one-third of farmers earned less than 10,000 BDT annually, indicating economic vulnerability. Rearing systems were primarily semi-intensive (67.3%), followed by free-range (24.1%) and intensive (8.5%), potentially influencing disease exposure [52]. Good knowledge of PPR was observed in 67.7% of farmers, with males and those aged 31–45 years demonstrating higher awareness and adoption of positive practices, particularly in Sylhet’s Haor regions [53]. These results underscore the importance of gender-sensitive, regionally tailored awareness and socio-economic support programs to enhance PPR control.

### Co-infections in PPRV-positive goats

A limited number of studies (4.54%) investigated co-infections in goats diagnosed with PPRV in Bangladesh, revealing a critical gap in syndromic research. The study involved 100 clinically suspected goats, of which 55% were confirmed PPRV-positive through RT-PCR. Among the confirmed cases, 58.2% exhibited co-infections with other pathogens [54]. The identified co-infections included *Klebsiella* spp. (n = 10), FMDV (n = 6), goat pox virus (n = 2), and *Mycobacterium* spp. indicative of tuberculosis (n = 2), confirmed using PCR and RT-PCR methodologies. Additionally, concurrent fascioliasis was observed in 12 goats based on clinical and parasitological examination.

A high prevalence (58.2%) of co-infections among PPRV-positive goats suggests that these findings underscore the high prevalence of co-infections in PPR-affected animals, likely due to the immunosuppressive effects of PPRV. The epidemiological complexity is further increased by the endemic presence of contagious caprine pleuropneumonia (CCPP) [55], caused by the bacterium *Mycoplasma capricolum* subsp. *capripneumoniae*, with reported seroprevalence (7.21%) and PCR positivity in lung samples (26.67%), along with identified risk factors such as age >18 months, female sex, large flock size, and poor body condition score [56]. This highlights clinical overlap with PPR, as diagnostic facilities in Bangladesh are limited, which can lead to misdiagnosis and underreporting. The frequent occurrence of bacterial, viral, and parasitic co-pathogens in infected goats, along with co-endemic diseases such as CCPP, may exacerbate morbidity, mask PPR outbreaks, and reduce post-vaccination immune responses, emphasizing the need for integrated disease surveillance and control frameworks.

### PPR vaccine efficacy and field performance in Bangladesh

Approximately 18.18% of studies evaluated the field efficacy of the locally developed PPR vaccine, “PPR VAC,” produced by the Livestock Research Institute, Mohakhali, Dhaka. The vaccine induced robust protective immunity in goats, lasting up to 12 months [51]. Another study reported a 62% seropositivity rate following administration of the conventional PPR vaccine [36], and no clinical or adverse effects were observed with PPR VAC administration [50]. A researcher concluded that a single annual vaccination with PPR VAC is effective for controlling PPR in the Chuadanga district [42].

The studies evaluated the locally developed PPR VAC vaccine, confirming its safety and year-long immunity under field conditions. “PPR VAC” produced by the Livestock Research Institute, Mohakhali, Dhaka, and imported vaccines showed post-vaccination seroconversion exceeding 91% in 2024, with division-level variation ranging from 87.96% to 96.19% [26]. Several studies lacked detailed reporting of study design and key variables, limiting meta-analytical synthesis. However, stricter inclusion criteria might have excluded valuable epidemiological insights. Importantly, data on the economic impact of PPR and the effectiveness of eradication efforts in Bangladesh remain scarce, as current seroconversion rates of PPR vaccine in 2025 were reported to be more than 75% in vaccinated small ruminants at the field level (sheep and goats) [57]. In Bangladesh, approximately 8–9.5 million PPR vaccine doses are administered annually; however, coverage remains uneven, exceeding 90% in targeted areas but remaining below 20% in many regions [58]. Sustained vaccination coverage of about 70–80% of the small ruminant population is required to achieve effective control and eradication under the strategy led by the Food and Agriculture Organization and the World Organization for Animal Health.

Wildlife, especially semi-domesticated ruminants, are largely excluded from vaccination programs, creating potential viral reservoirs. There is no information and research on vaccination and vaccine efficacy in wild and semi-wild ruminants. The absence of DIVA-compatible vaccines limits accurate post-vaccination surveillance in Bangladesh. DIVA strategies could enhance risk-based monitoring, improve outbreak attribution, and support verification of PPR freedom during eradication efforts. These represent an important area for future research.

## CONCLUSION

This systematic review synthesizes evidence from 22 studies and demonstrates that PPR remains endemic in Bangladesh with substantial heterogeneity in study design, geographic coverage, and reported outcomes. The disease burden shows marked regional variation, with prevalence ranging from 2.18% to 42.26%, and consistently higher susceptibility in goats compared to sheep. Seroprevalence findings indicate widespread circulation of PPRV across different regions, with the highest seropositivity observed in buffalo (42.36%) despite limited data on large ruminants. Molecular investigations confirm the exclusive circulation of Lineage IV PPRV in Bangladesh, with strong genetic similarity to strains from neighboring countries, highlighting the role of transboundary transmission. Diagnostic practices rely primarily on enzyme-linked immunosorbent assay and RT-PCR, while advanced molecular tools remain underutilized. Host-related factors such as age and breed, along with environmental drivers including seasonality and animal movement (p = 0.03), play significant roles in disease occurrence. Co-infections are frequently reported (58.2%), indicating the complexity of clinical presentation and the influence of immunosuppression on disease progression. The locally produced PPR VAC demonstrates strong immunogenicity, with protection lasting up to 12 months and seroconversion exceeding 91%, although vaccination coverage remains uneven across regions.

From a practical perspective, these findings emphasize the urgent need to strengthen integrated surveillance systems, particularly in underrepresented regions such as Sylhet and Barishal. Improved biosecurity in animal markets and slaughterhouses is essential to reduce transmission. Expansion of vaccination programs with consistent coverage of at least 70–80% of the small ruminant population is critical for effective control. The incorporation of rapid molecular diagnostics at field level would enhance early detection and outbreak response. In addition, addressing co-infections through integrated disease management strategies is necessary to improve clinical outcomes and vaccine performance.

A major strength of this review lies in its comprehensive synthesis of epidemiological, diagnostic, molecular, and socio-economic evidence specific to Bangladesh, providing a consolidated understanding of PPR dynamics. The inclusion of multiple study types and regions enhances the robustness of the findings. However, several limitations should be acknowledged. The available studies exhibit significant heterogeneity in design, sampling strategies, and diagnostic approaches, which limits comparability. Geographic clustering of studies and underrepresentation of wildlife and large ruminants restrict a complete epidemiological picture. In addition, limited reporting on morbidity, mortality, and economic impact constrains a full assessment of disease burden. The lack of standardized methodologies across studies further limits the potential for quantitative synthesis.

Future research should prioritize multi-species surveillance, including wildlife reservoirs, to better understand transmission dynamics. There is a critical need for the adoption of advanced molecular diagnostics and the development of DIVA-compatible vaccines to improve post-vaccination monitoring. Longitudinal and nationwide studies evaluating vaccine effectiveness under field conditions are essential. Furthermore, research integrating socio-economic factors, farmer behavior, and policy interventions will be vital for designing sustainable control strategies. Strengthening cross-border collaboration and regional data sharing will also be important given the transboundary nature of PPRV.

In conclusion, achieving PPR eradication in Bangladesh by 2030 will require a coordinated, evidence-based approach integrating vaccination, surveillance, diagnostics, and biosecurity measures. Addressing current knowledge gaps, ensuring equitable vaccine coverage, and adopting innovative diagnostic and monitoring tools will be critical to meeting national and global eradication targets and ensuring long-term resilience of the livestock sector.

## DATA AVAILABILITY

The data generated during the study are included in the manuscript.

## AUTHORS’ CONTRIBUTIONS

SA: Conceived and designed the study, performed literature search and screening, data extraction, and drafted the manuscript. SBJ: Contributed to study design, data interpretation, and critical revision of the manuscript. MR: Participated in data collection, analysis, and manuscript preparation. SCB: Contributed to methodology development and validation of data. SM: Assisted in data interpretation and manuscript revision. MMK: Study conception and design supervised the research, and critically revised the manuscript for important intellectual content. All authors have read and approved the final manuscript and agree to be accountable for all aspects of the work.
